# Kinetic Study of Metalloporphyrin-Based Biomimetic Oxidation of Drugs Using One-Shot and Continuous Oxidizing Agent Addition Modes

**DOI:** 10.1021/acs.jpcb.6c00851

**Published:** 2026-04-28

**Authors:** Balázs Csillag, Ernák Ferenc Várda, Anna Kamilla Kis, Zsombor Máté Hámor, Zsombor Márton Mohácsi, Anna Vincze, András Dénes Marton, Balázs Decsi, Diána Balogh-Weiser, Balázs Volk, Arash Mirzahosseini, György Tibor Balogh

**Affiliations:** 1 Department of Pharmaceutical Chemistry, 37637 Semmelweis University, Budapest H-1092, Hungary; 2 Center for Pharmacology and Drug Research & Development, 37637 Semmelweis University, Budapest H-1085, Hungary; 3 Department of Chemical and Environmental Process Engineering, 61810 Budapest University of Technology and Economics, Budapest H-1111, Hungary; 4 Ambimass LLC, Budapest H-1031, Hungary; 5 Department of Physical Chemistry and Materials Science, 61810 Budapest University of Technology and Economics, Budapest H-1111, Hungary; 6 Department of Organic Chemistry and Technology, 61810 Budapest University of Technology and Economics, Budapest H-1111, Hungary; 7 Spinsplit LLC, Vecsés H-2220, Hungary; 8 Directorate of Drug Substance Development, 58965 Egis Pharmaceuticals PLC, Budapest H-1106, Hungary

## Abstract

A systematic kinetic study of metalloporphyrin-catalyzed biomimetic oxidation was performed to elucidate the relationships between reaction conditions, catalyst behavior, and oxidation rates of substrates. Using FeTPPS as a model metalloporphyrin catalyst, a preliminary screening established the optimal amount of oxidizing agent and mode of addition. Continuous administration of 10 equiv of oxidant produced gradual, measurable reaction progress suitable for quantitative kinetic modeling. A mechanistic kinetic model was developed based on a system of ordinary differential equations describing catalyst activation, substrate oxidation, and catalyst deactivation, and it was successfully fitted to experimental data obtained under both one-shot and continuous oxidant addition. The approach was extended to multiple substrates and pH conditions. In specific cases, inclusion of a noncatalytic oxidation term improved the fit of the model, indicating a minor parallel oxidation pathway. The substrate-specific rate constants derived from the model showed good proportionality with literature intrinsic clearance values determined from liver microsomal assays, which represent the gold standard for intrinsic clearance estimation, thereby supporting the physiological relevance of the biomimetic system. Overall, the combined experimental and computational framework provides a quantitative basis for interpreting biomimetic oxidation kinetics and establishes a mechanistic bridge between enzymatic catalysis and pharmacokinetic clearance processes.

## Graphical abstract

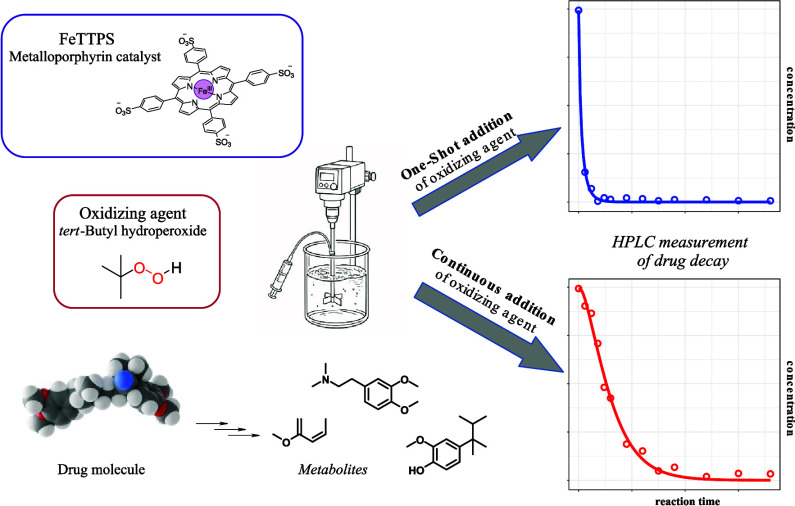

## 1 Introduction

The oxidative metabolism of pharmaceuticals plays a crucial role in determining their pharmacological activity and bioavailability. In biological systems, these transformations are mostly catalyzed by cytochrome P450, which incorporates oxygen into organic molecules via monooxygenase-type processes.^1^ Understanding these oxidative pathways is essential for predicting potential metabolites, toxicity and the environmental fate of pharmaceutical impurities.^2^ However, *in vivo* or *in vitro* enzymatic studies are often limited by the complexity of biological systems and high experimental costs.^3^ To address these limitations, biomimetic oxidation systems have been developed as simplified chemical models that reproduce the reactivity of enzymatic oxidation processes. Among these, metalloporphyrin-based catalysts have been the most popular, because of their ability to mimic the active site of cytochrome P450 enzymes.^4^ Iron­(III) porphyrins demonstrated high efficiency in catalyzing specific oxidation reactions due to their compatibility with common oxidants such as hydrogen peroxide or *tert*-butyl hydroperoxide (tBuOOH).^5^

By using metalloporphyrin catalysts immobilized on solid magnetic nanoparticles (MNPs) as carriers we combine the catalytic advantages of a homogeneous system with easier separation or even recyclability.^6,7^ Also, the broader field of nanocatalysis has been highlighted as the cornerstone of green chemistry, because of the ability of nanocatalysts to enhance reactivity and selectivity.^8^ Despite extensive research on biomimetic oxidation systems, the kinetic dimensions of these reactions are still inadequately comprehended. Kinetic analysis provides critical insight into the correlation between the parameters that are the most significant in determining the selectivity and efficiency of oxidation processes, such as substrate reactivity, catalyst activity and oxidant concentration.^9^ The mode of oxidant addition, whether introduced as a one-shot or continuously supplied over time, can significantly affect both the reaction rate and the selectivity profile. While an excess of oxidant can lead to enhanced catalyst degradation and nonselective oxidation, controlled oxidant dosing can reduce side reactions and more closely mimic enzymatic catalysis.^10^ In this context, continuous oxidant addition is markedly superior to one-shot dosing, as it preserves catalyst activity, enables time-resolved kinetic analysis, and yields reaction profiles that are more directly comparable to enzymatic turnover. This research investigates the kinetic behavior of biomimetic drug oxidation catalyzed by a magnetically separable metalloporphyrin system, comparing two oxidant-delivery approaches: one-shot and continuous oxidant addition. Reaction progress was monitored using LC-MS analysis.

## 2 Methods

### 2.1 Preparation of Metalloporphyrin Magnetic Nanoparticles

3-aminopropyl-functionalized MNP (130 mg) and mixture of methanol and acetate buffer (2 mL, 4:1 (*V*/*V*), pH 4.5, 64 mmol L^–1^) were added, then suspended by sonication for 15 min (at RT, with full power at 45 kHz, Emmi 20HC ultrasonic bath, Emag AG, Mörfelden-Walldorf, Germany). Solution of FeTPPS [5,10,15,20-tetrakis­(4-sulfonatophenyl)­iron­(II) porphyrin purchased from Frontier Scientific (Logan, UT, USA)] (3 mL, 0.3 mg mL^–1^, dissolved in the mixture of methanol and acetate buffer was added to the suspension. The mixture was shaken for another 10 min (at RT, 800 rpm, Vibramax 100, Heidolph NA Llc, Schwabach, Germany), then the nanoparticles were magnetically isolated with a N35 permanent magnet. From the residual binding solution sample was taken (1 mL), to determine the immobilization yield of the FeTPPS porphyrin. The separated nanoparticles were washed two times with methanol, then dried in a vacuum drying cabinet for 2 h (at 100 mbar, in a Binder VDL 23 vacuum drying chamber, Binder GmbH, Tuttlingen, Germany).

### 2.2 Experiments with One-Shot Oxidant Addition

First, we prepared a 10 mmol L^–1^ stock solution of the chosen pharmaceuticals in DMSO (dimethyl sulfoxide). Then we prepared a 10 mg (MNP)­FeTPPS suspension in 5 mL of buffer, which was sonicated for 10 min in order to disperse the catalyst in the buffer and break up the nanoparticle aggregations thus maximizing the reaction surface. Next, the experiment was assembled in a half diffusion cell: the sonicated catalyst, 4.5 mL of buffer and 0.5 mL of stock solution were added to obtain a 500 μmol L^–1^ initial concentration of the chosen substance. The experiment was initiated by adding 7 μL of *tert*-butyl hydroperoxide oxidizing agent to the half diffusion cell. During the 1 h experiment, 150 μL samples were collected at 2, 4, 6, 8, 10, 15, 20, 25, 30, 40, 50, and 60 min. The catalyst was then separated from each sample using a permanent magnet before transferring 100 μL of the solution into a separate vial. The experiments were conducted at 37 °C with continuous stirring, at two different pH levels with each drug in triplicates. The samples were then analyzed with LC-MS.

### 2.3 Experiments with Continuous Oxidant Addition

The stock solution of the chosen pharmaceuticals and (MNP)­FeTPPS suspension was prepared as described before. The experiment was assembled in a half diffusion cell: the sonicated catalyst, 4.975 mL of buffer and 0.525 mL of stock solution were added to obtain a 500 μmol L^–1^ initial concentration of the chosen substance. From this suspension 1 mL was collected (after magnetic separation) and 14 μL of tBuOOH was added to the clarified supernatant. Then, this solution was loaded into a 2.5 mL syringe and the experiment was initiated by dispensing the syringe contents through a capillary back into the reaction mixture using a pump. The flow rate was set to 0.5 mL h^–1^, ensuring that by the end of the 1 h reaction 0.5 mL of the syringe’s content (containing 7 μL of tBuOOH) was reintroduced into the half-diffusion cell. During the 1 h experiment 150 μL samples were collected at the same time points as described above. The catalyst was then separated from each sample using a permanent magnet before transferring 100 μL of the solution into a separate vial. The experiments were conducted at 37 °C with continuous stirring, at two different pH levels with each drug in triplicates. The samples were then analyzed with LC-MS.

### 2.4 Analytical Measurements

The analysis of the samples was carried out with LC-MS. A Waters 2795 HPLC with a Waters 2487 DAD detector was coupled with Micromass Quattro Ultima TMPt quadrupole mass spectrometer equipped with an ESI source. Measurements were conducted using a Waters XSelect C18 (150 mm × 4.6 mm; 3.5 μm) column at a temperature of 40 °C and with an eluent flow rate of 1 mL min^–1^. Eluent A consisted of Milli-Q water containing 0.1% (*V*/*V*) formic acid, while eluent B was a mixture of acetonitrile and Milli-Q water (95:5, *V*/*V*) also containing 0.1% (*V*/*V*) formic acid. The chromatographic separation was achieved using the following linear gradient: 0–7 min 5–100% B; held at 100% for 1 min; followed by a 2 min-long re-equilibration to the initial composition (5% B, 95% A) at 8.01 min. The injection volume for all samples was 5 μL. Chromatic profiles were recorded at dual wavelengths of 240 and 270 nm. The operating parameters of the mass spectrometer were as follows: a mass range of *m*/*z* 150–600, nitrogen gas flow rate of 350 L h^–1^, temperature of 350 °C and pressure of 6 bar. The quadrupole temperature was set to 120 °C with a capillary voltage of 2.5 kV and a fragmentor voltage of 60 V.

### 2.5 Statistical Analysis

Kinetic model fitting and statistical evaluation were performed in the R environment (v4.5.1).^11^ Experimental data were analyzed separately for each compound, pH, and oxidizing agent addition mode. Model parameters (*k*_1_, *k*_2_, *k*_3_, and *k*_4_ where applicable) were estimated by nonlinear least-squares minimization using the Levenberg–Marquardt algorithm as implemented in the nls.lm­() function (package minpack.lm^12^). The objective function minimized the sum of squared residuals between experimental chromatographic peak areas and model-predicted substrate concentrations obtained from numerical integration of the ordinary differential equations (ODEs) using the ode­() solver from the deSolve package.^13^ Initial parameter estimates were first guessed by quick manual inspection using the ode23­() function from the pracma package,^14^ and then optimized iteratively. The oxidizer feed term (δ) was adjusted according to the experimental oxidant excess and maximum reaction time. The initial substrate concentration was refined by minimizing the residual standard deviation across a grid of 20 perturbed values within ± 3 global standard deviations of the experimental mean, ensuring stable convergence and model identifiability. Goodness of fit was assessed by the adjusted coefficient of determination (*R*_*adj*_^2^), as well as by the Akaike Information Criterion (AIC). Parameter uncertainty was propagated by parametric Monte Carlo simulation. A total of 100 parameter sets were generated by random sampling from normal distributions centered at the fitted values with standard deviations equal to their estimated standard errors. For each parameter set, the ODE system was resolved, and simulated concentration–time curves were obtained. From these, the 95% confidence interval (CI) bands were constructed as the 2.5 th and 97.5 th percentiles of the simulated trajectories. The resulting simulated envelopes were plotted together with the experimental data and best-fit curves to visualize the agreement between the experimental kinetics and the model. All plots were generated with ggplot,^15^ and all numeric and graphical analyses were performed in RStudio Version: 2025.09.0 + 387 (Posit, Boston, MA, USA).

## 3 Results

### 3.1 Preliminary Study

To establish the kinetic range and optimal experimental conditions for the biomimetic oxidation mediated by the metalloporphyrin catalyst, a preliminary screening was performed. This mini-screen assessed several key parameters influencing the oxidation process: the excess of oxidizing agent, reaction time, pH, and the mode of oxidant addition (either single-dose one-shot addition or continuous linear delivery). Verapamil was selected as the model substrate, as this compound had previously shown favorable and reproducible behavior in preliminary conversion screens (data not shown). An almost instantaneous oxidation was observed with one-shot addition of the oxidizing agent, preventing meaningful kinetic analysis. In contrast, continuous oxidant addition yielded a gradual reaction profile that allowed the oxidation kinetics to be monitored over time; only under these conditions were the reaction rates sufficiently time-resolved to permit reliable kinetic modeling and parameter estimation. The influence of pH was not evaluated in the preliminary study; a pH with reliable metalloporphyrin stability and performance was chosen based on mechanistic knowledge of the catalyst. As can be seen from Figure 1, the optimal conditions were determined to be the continuous addition of 10 equiv of the oxidizing agent, which provided the most consistent and interpretable kinetic profiles.

**Figure 1:**
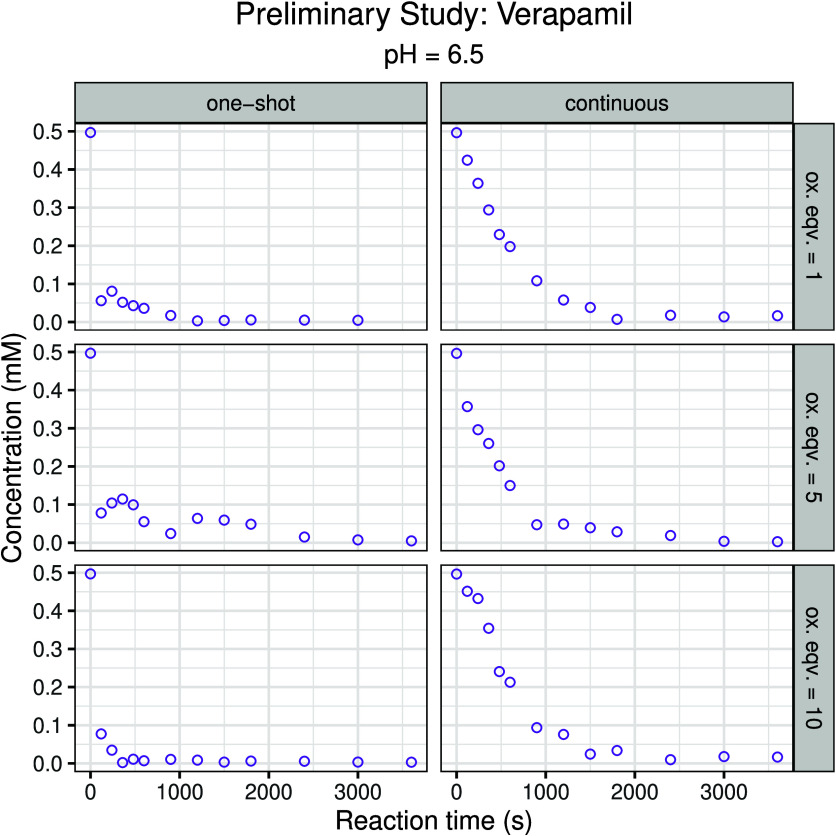
Preliminary screening of the biomimetic oxidation of verapamil catalyzed by the metalloporphyrin complex. Comparison of oxidant addition modes (one-shot vs continuous) illustrating the effect on reaction kinetics.

### 3.2 Kinetic Analysis

Following the preliminary screening, the kinetic behavior of the biomimetic oxidation system was quantitatively modeled. The reaction scheme was constructed based on three key processes: (i) activation of the FeTPPS catalyst by the oxidizing agent, (ii) oxidation of the substrate leading to regeneration of FeTPPS, and (iii) gradual inactivation of FeTPPS through oxidative degradation of catalyst. It is important to note that these processes (especially step three) are mechanistically complex and likely involve multiple parallel pathways of different kinetic orders; therefore, in the present model, the corresponding rate constant (*k*_3_) was treated as an overall apparent descriptor of FeTPPS deactivation. Furthermore, in the following reaction schemes only the species of kinetic interest are depicted; side-products (e.g., reduction products of tBuOOH) are omitted.

$$\begin{array}{ll} \mathrm{FeTPPS}+t\mathrm{BuOOH} & \overset{k_{1}}{\to }{\mathrm{FeTPPS}}^{*}\\ \mathrm{Substrate}+{\mathrm{FeTPPS}}^{*} & \overset{k_{2}}{\to }\mathrm{Metabolite}+\mathrm{FeTPPS}\\ \mathrm{FeTPPS}+t\mathrm{BuOOH} & \overset{k_{3}}{\to }{\mathrm{FeTPPS}}^{0} \end{array}$$ 1

The individual reaction steps were translated into rate laws, forming a system of ordinary differential equations (ODEs) that describe the time-dependent concentration changes of all reactive species.

$$\begin{array}{ll} \frac{d\left[\mathrm{FeTPPS}\right]}{dt} & =-\left(k_{1}+k_{3}\right)\left[\mathrm{FeTPPS}\right]\left[\mathrm{tBuOOH}\right]+k_{2}\left[\mathrm{Substrate}\right]\left[{\mathrm{FeTPPS}}^{*}\right]\\ \frac{d\left[{\mathrm{FeTPPS}}^{*}\right]}{dt} & =k_{1}\left[\mathrm{FeTPPS}\right]\left[t\mathrm{BuOOH}\right]-k_{2}\left[\mathrm{Substrate}\right]\left[{\mathrm{FeTPPS}}^{*}\right]\\ \frac{d\left[t\mathrm{BuOOH}\right]}{dt} & =-\left(k_{1}+k_{3}\right)\left[\mathrm{FeTPPS}\right]\left[\mathrm{tBuOOH}\right]\\ \frac{d\left[\mathrm{Substrate}\right]}{dt} & =-k_{2}\left[\mathrm{Substrate}\right]\left[{\mathrm{FeTPPS}}^{*}\right] \end{array}$$ 2

In the case of continuous oxidant addition, the ODE system was modified by inclusion of a linear term accounting for the gradual input of the oxidizing agent over time (eq 3 instead of [Disp-formula eq2]c).

$$\frac{d\left[t\mathrm{BuOOH}\right]}{dt}=-\left(k_{1}+k_{3}\right)\left[\mathrm{FeTPPS}\right]\left[t\mathrm{BuOOH}\right]+\delta$$ 3

The initial concentrations of the species were defined based on the experimental setup, using the specified number of oxidant equivalents (eqv). Accordingly, the initial conditions were set as follows:

$$\begin{array}{ll} {}[\mathrm{FeTPPS}{]}_{t=0} & =\frac{[\mathrm{Substrate}{]}_{t=0}}{\mathrm{eqv}}\\ {\left[{\mathrm{FeTPPS}}^{*}\right]}_{t=0} & =0\\ {}[t\mathrm{BuOOH}{]}_{t=0} & =\mathrm{eqv}\cdot [\mathrm{Substrate}{]}_{t=0} \end{array}$$ 4

For the case of continuous oxidant addition, eq 4c is modified as follows, with the oxidant initially absent and introduced linearly over time. The corresponding feed term δ is defined by the experimental oxidant delivery rate:

$$\begin{array}{ll} {}[t\mathrm{BuOOH}{]}_{t=0} & =0\\ \delta & =\frac{\mathrm{eqv}\cdot [\mathrm{Substrate}{]}_{t=0}}{t_{\max}} \end{array}$$ 5

The model was numerically solved and fitted to the experimental kinetic data, yielding excellent agreement between calculated and observed values. As illustrated in Figure 2, the fitted curves accurately reproduce the experimental trends, confirming that the proposed mechanistic model captures the essential features of the biomimetic oxidation process. In the kinetic plots, the observed signal is presented as HPLC peak area rather than concentration, because simple rescaling does not affect parameter estimation, while reliable conversion of metabolite peak areas to concentrations was not possible due to the unknown molar response coefficients of the metabolites.

**Figure 2:**
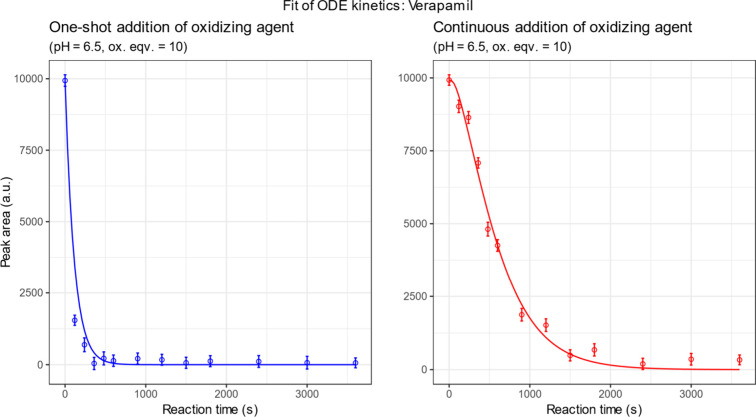
Kinetic modeling of the biomimetic oxidation of verapamil catalyzed by FeTPPS. Experimental data (symbols) and fitted model curves (lines) obtained from numerical solutions of the system of ordinary differential equations. The model incorporates catalyst activation, substrate oxidation, and catalyst inactivation steps, and includes a linear oxidant feed term for continuous addition.

### 3.3 Extensive Study

The kinetic modeling framework was subsequently applied to a broader series of compounds in order to evaluate the generality of the proposed mechanism and to identify compound-dependent trends in biomimetic oxidation behavior. In total, six additional substrates were investigated under various pH conditions using the same metalloporphyrin catalytic system and optimized continuous oxidant addition protocol established in the preliminary study.

Figure 3 presents the chemical structures of all substrates included in this extended data set, along with their corresponding oxidation products identified by chromatographic and mass spectrometric analysis. The product profiles were consistent with mono- or multihydroxylated or cleaved metabolites expected for oxidative transformations mediated by FeTPPS.

**Figure 3:**
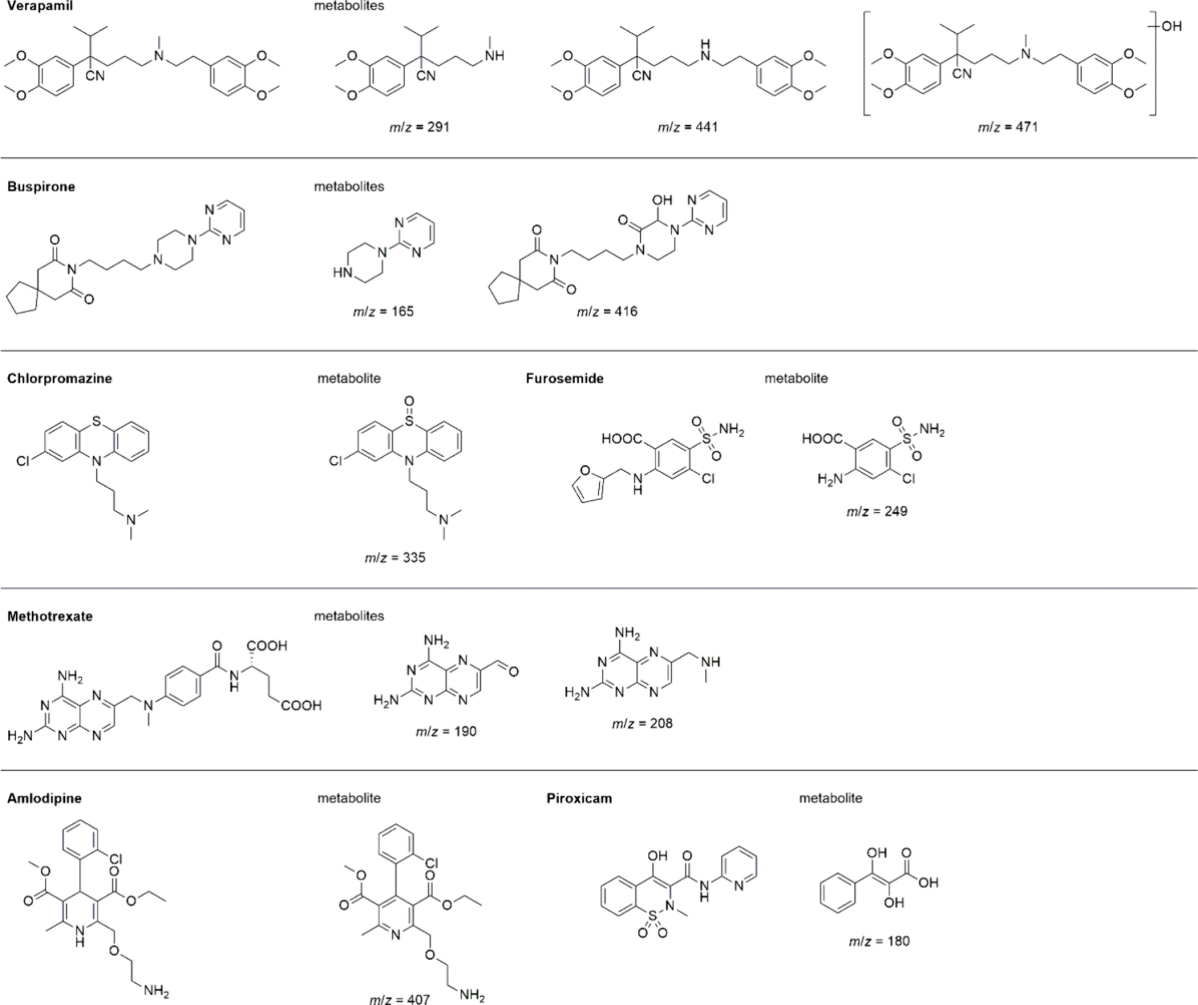
Chemical structures of the compounds investigated in the extended biomimetic oxidation study and their corresponding oxidation products.

The following sections provide a detailed account of the experimental kinetic results obtained for each compound, together with the corresponding model fits and derived rate parameters. These comparative analyses allow the evaluation of structural and electronic factors influencing the biomimetic oxidation kinetics across the studied compound series.

Figure 4 illustrates the experimental results obtained for verapamil oxidation under various pH conditions. The kinetic model could be successfully fitted only at pH 4.5 and 6.5, as under more strongly acidic conditions the metalloporphyrin oxidation mechanism deviated from the modeled pathway and did not yield meaningful kinetic parameters. Among the two pH values modeled, distinct differences in reaction behavior were observed. At pH 6.5, the kinetic profile displayed a more pronounced substrate conversion and a superior model fit, reflected by closer agreement between experimental and calculated data and by a narrower 95% confidence interval band. The numerical solution of the ODE system also provides concentration–time profiles for all participating species. Accordingly, in addition to the experimental and modeled curves of the substrate, the simulated concentration profiles of the metalloporphyrin species and the oxidizing agent are also depicted. The curve corresponding to FeTPPS shows a rapid initial decrease due to its conversion to the activated FeTPPS* form. The concentration of the activated species subsequently declines following an initial rise, reflecting the gradual degradation of FeTPPS in the presence of excess oxidizing agent.

**Figure 4:**
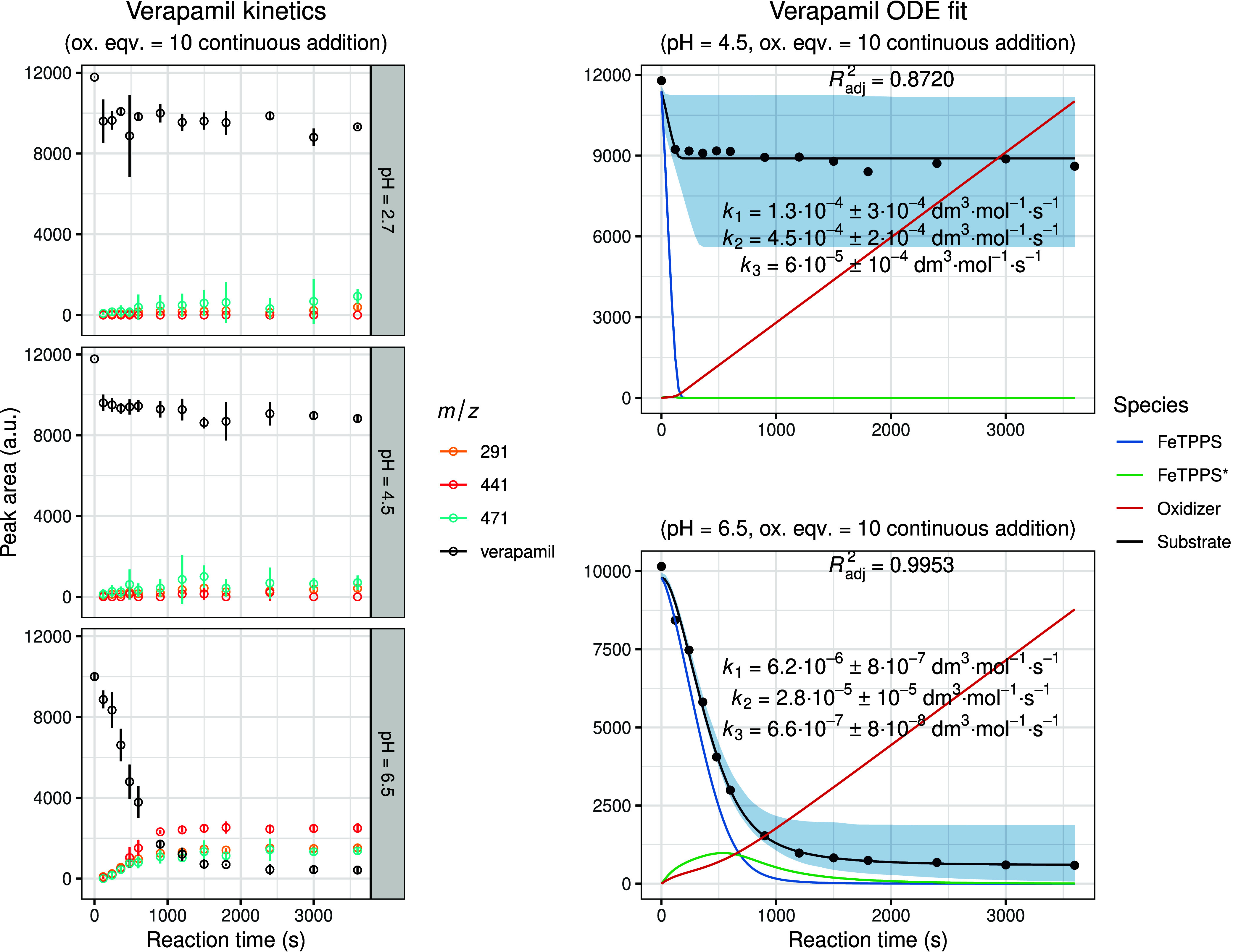
Experimental and modeled biomimetic oxidation kinetics of verapamil. The left panel shows the experimental kinetic data obtained from three independent parallel experiments (mean ± SD), illustrating the time-dependent change in chromatographic peak areas for the substrate and identified oxidation metabolites. The right panel presents the corresponding fitted kinetic model together with simulated 95% confidence intervals (shaded regions). The calculated concentration–time profiles of FeTPPS and activated FeTPPS* (scaled by × 10), as well as the oxidizing agent (scaled by × 0.1), are included to visualize the dynamic behavior of all reactive species within the system.

Figure 5 depicts the experimental and modeled results for buspirone oxidation, which exhibited overall similar kinetic characteristics to those observed for verapamil. However, a key difference was noted in the extent of substrate conversion: the substrate concentration did not decay to near zero. This incomplete conversion suggests a gradual loss of FeTPPS activity during oxidant addition, likely due to catalyst degradation, which ultimately brings the catalytic cycle to a halt.

**Figure 5:**
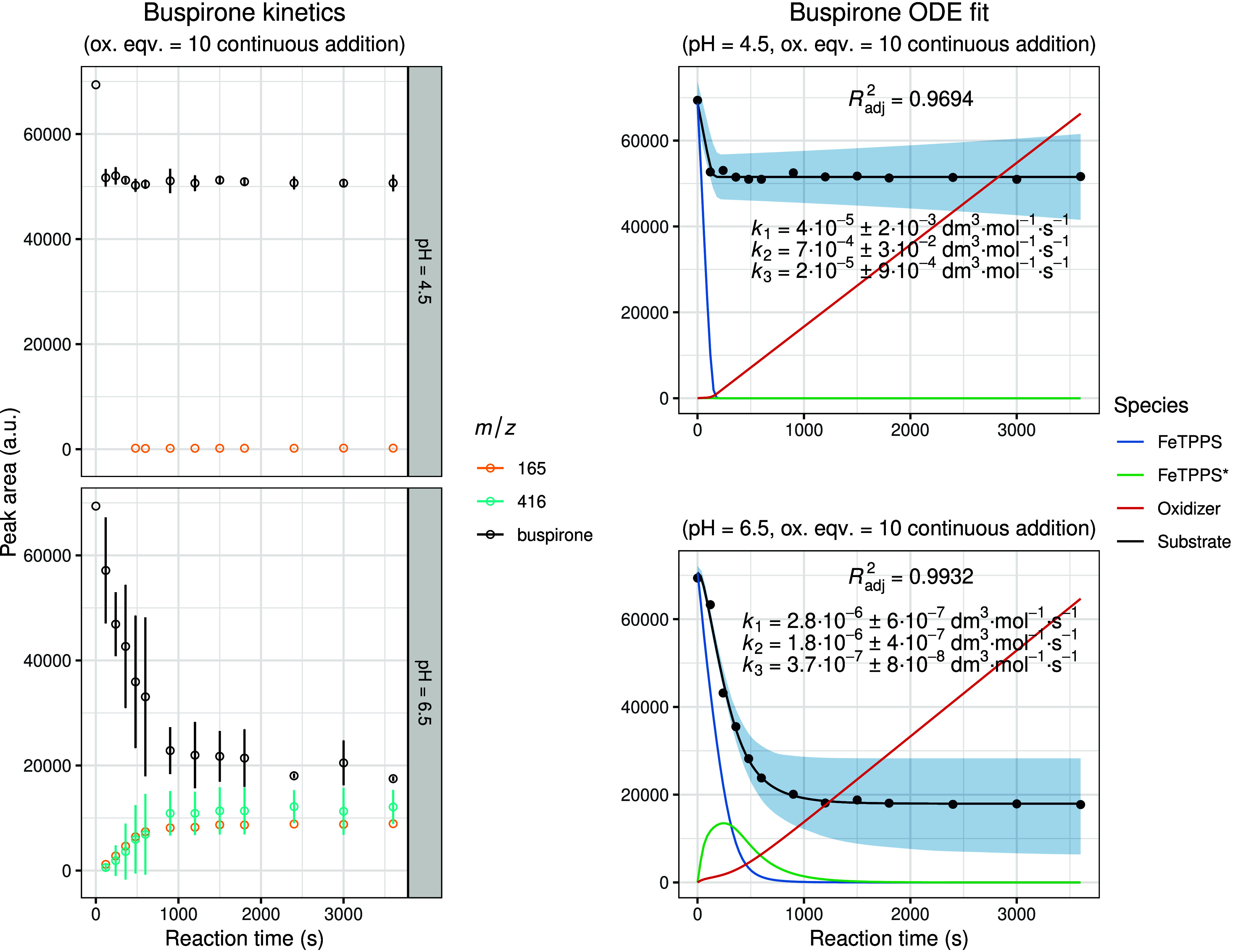
Experimental and modeled biomimetic oxidation kinetics of buspirone. The left panel shows the experimental kinetic data obtained from three independent parallel experiments (mean ± SD), illustrating the time-dependent change in chromatographic peak areas for the substrate and identified oxidation metabolites. The right panel presents the corresponding fitted kinetic model together with simulated 95% confidence intervals (shaded regions). The calculated concentration–time profiles of FeTPPS and activated FeTPPS* (scaled by × 10), as well as the oxidizing agent (scaled by × 0.1), are included to visualize the dynamic behavior of all reactive species within the system.

Figure 6 depicts the experimental and modeled results for chlorpromazine oxidation. In this case, the kinetic profile showed a striking deviation from the previously analyzed compounds: the decay of the substrate consistently followed a biphasic, two-step curve that could not be adequately described by the initial kinetic model. To account for this behavior, the model was extended to include a spontaneous (noncatalytic) oxidation pathway (as implied by experiments containing no metalloporphyrin) in which the substrate reacts directly with the oxidizing agent in the absence of FeTPPS.

**Figure 6:**
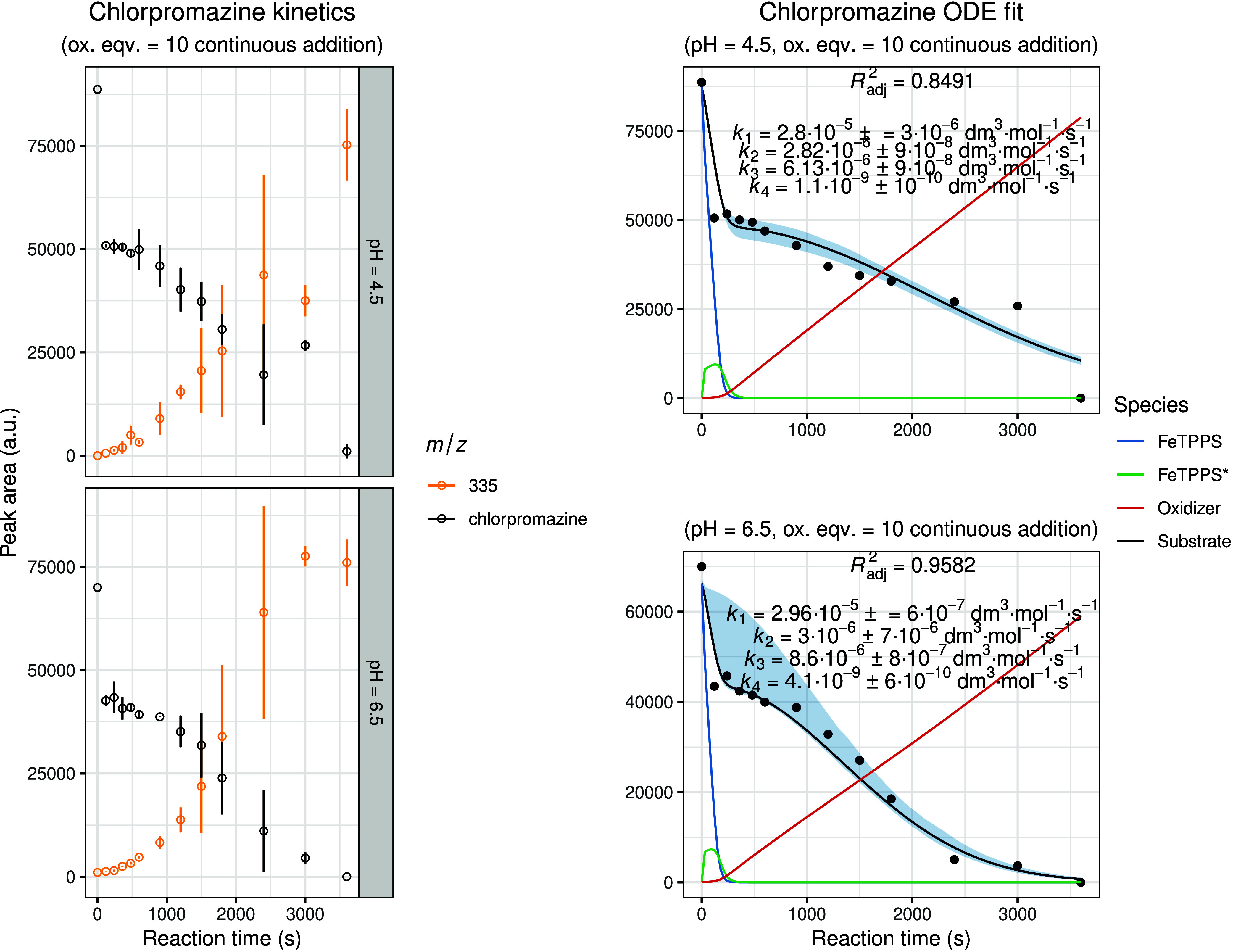
Experimental and modeled biomimetic oxidation kinetics of chlorpromazine. The left panel shows the experimental kinetic data obtained from three independent parallel experiments (mean ± SD), illustrating the time-dependent change in chromatographic peak areas for the substrate and identified oxidation metabolites. The right panel presents the corresponding fitted kinetic model together with simulated 95% confidence intervals (shaded regions). The calculated concentration–time profiles of FeTPPS and activated FeTPPS* (scaled by × 10), as well as the oxidizing agent (scaled by × 0.1), are included to visualize the dynamic behavior of all reactive species within the system.

Accordingly, eq 2c,d was modified as follows:

$$\begin{array}{ll} \frac{d\left[t\mathrm{BuOOH}\right]}{dt} & =-\left(k_{1}+k_{3}\right)\left[\mathrm{FeTPPS}\right]\left[t\mathrm{BuOOH}\right]-k_{4}\left[\mathrm{Substrate}\right]\left[\mathrm{tBuOOH}\right]+\delta \\ \frac{d\left[\mathrm{Substrate}\right]}{dt} & =-k_{2}\left[\mathrm{Substrate}\right]\left[{\mathrm{FeTPPS}}^{*}\right]-k_{4}\left[\mathrm{Substrate}\right]\left[\mathrm{tBuOOH}\right] \end{array}$$ 6

The resulting fits demonstrated excellent agreement with the experimental data, successfully capturing the two-step decay behavior through inclusion of a minor but measurable noncatalytic oxidation pathway. To verify the general applicability of this extended model, all other kinetic profiles, including those previously described, were re-evaluated using the same approach. In cases where incorporation of the noncatalytic term did not improve the fit or where the estimated *k*_4_ contribution was negligible, the original catalytic model was retained in line with the principle of parsimony.

Figures 7–10 summarize the experimental and modeled results for the oxidation of furosemide, methotrexate, amlodipine, and piroxicam under the optimized biomimetic conditions. Overall, the obtained kinetic profiles were consistent with those observed for the previously studied compounds, indicating comparable mechanistic behavior within the series. The only exception was methotrexate at pH 4.5, where the experimental data did not permit a meaningful kinetic fit, likely due to altered reaction kinetics or instability of the catalyst-substrate complex under these conditions. In instances where inclusion of the extended model (incorporating the noncatalytic oxidation term, *k*_4_) was necessary to achieve an improved fit, the corresponding *k*_4_ parameter and simulated curve are shown in the figures. For all other cases, the standard catalytic model was sufficient to accurately describe the experimental data.

**Figure 7:**
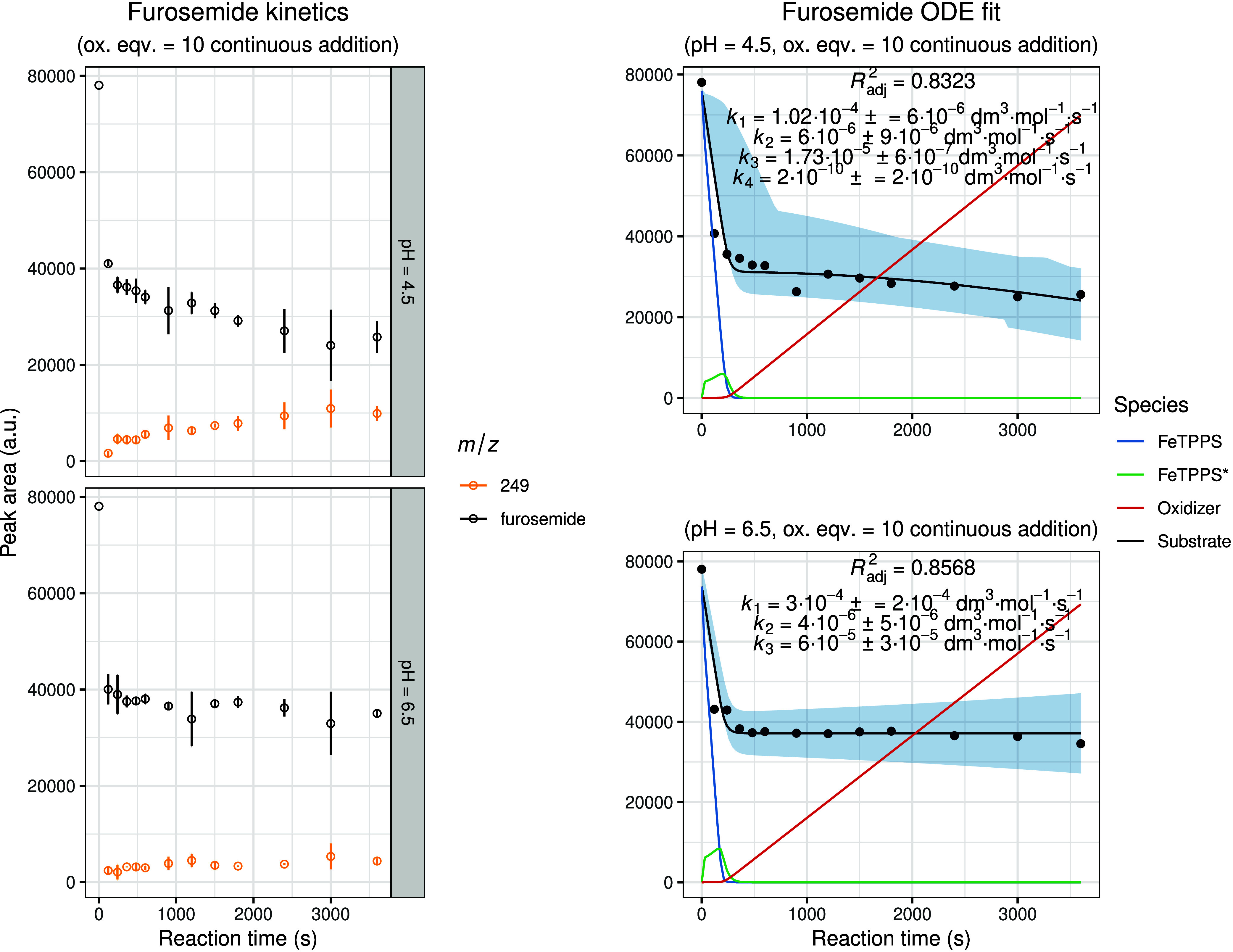
Experimental and modeled biomimetic oxidation kinetics of furosemide. The left panel shows the experimental kinetic data obtained from three independent parallel experiments (mean ± SD), illustrating the time-dependent change in chromatographic peak areas for the substrate and identified oxidation metabolites. The right panel presents the corresponding fitted kinetic model together with simulated 95% confidence intervals (shaded regions). The calculated concentration–time profiles of FeTPPS and activated FeTPPS* (scaled by × 10), as well as the oxidizing agent (scaled by × 0.1), are included to visualize the dynamic behavior of all reactive species within the system.

**Figure 8:**
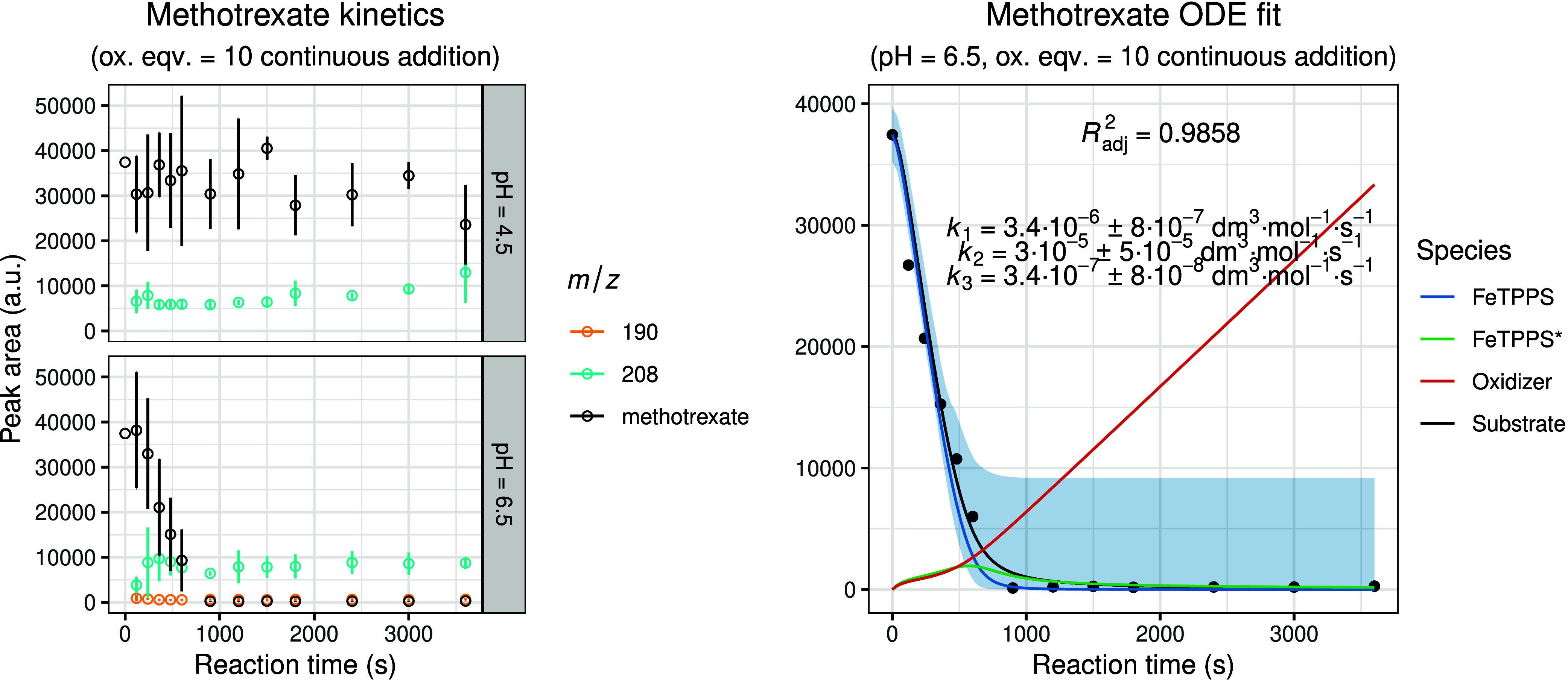
Experimental and modeled biomimetic oxidation kinetics of methotrexate. The left panel shows the experimental kinetic data obtained from three independent parallel experiments (mean ± SD), illustrating the time-dependent change in chromatographic peak areas for the substrate and identified oxidation metabolites. The right panel presents the corresponding fitted kinetic model together with simulated 95% confidence intervals (shaded regions). The calculated concentration–time profiles of FeTPPS and activated FeTPPS* (scaled by × 10), as well as the oxidizing agent (scaled by × 0.1), are included to visualize the dynamic behavior of all reactive species within the system.

**Figure 9:**
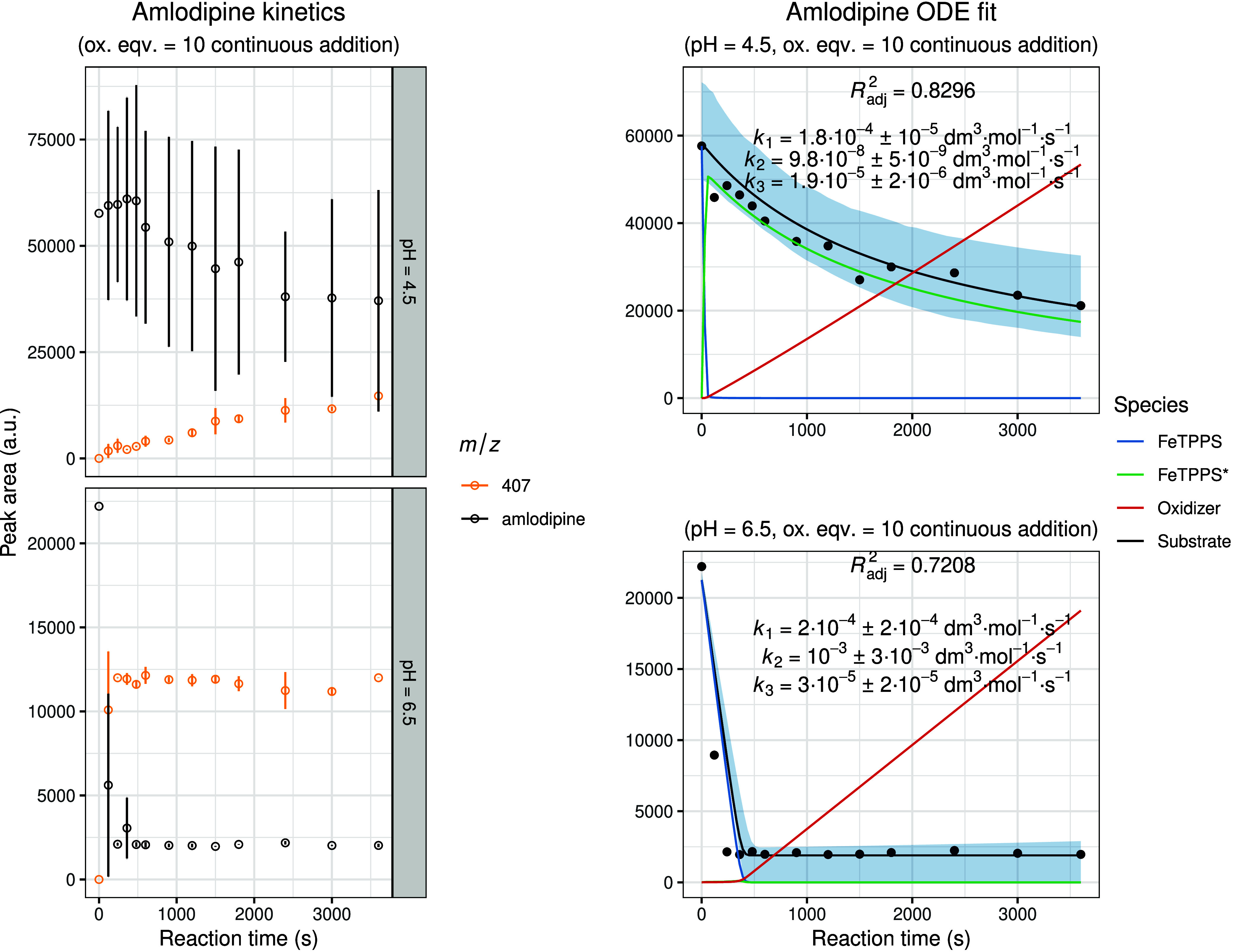
Experimental and modeled biomimetic oxidation kinetics of amlodipine. The left panel shows the experimental kinetic data obtained from three independent parallel experiments (mean ± SD), illustrating the time-dependent change in chromatographic peak areas for the substrate and identified oxidation metabolites. The right panel presents the corresponding fitted kinetic model together with simulated 95% confidence intervals (shaded regions). The calculated concentration–time profiles of FeTPPS and activated FeTPPS* (scaled by × 10), as well as the oxidizing agent (scaled by × 0.1), are included to visualize the dynamic behavior of all reactive species within the system.

**Figure 10:**
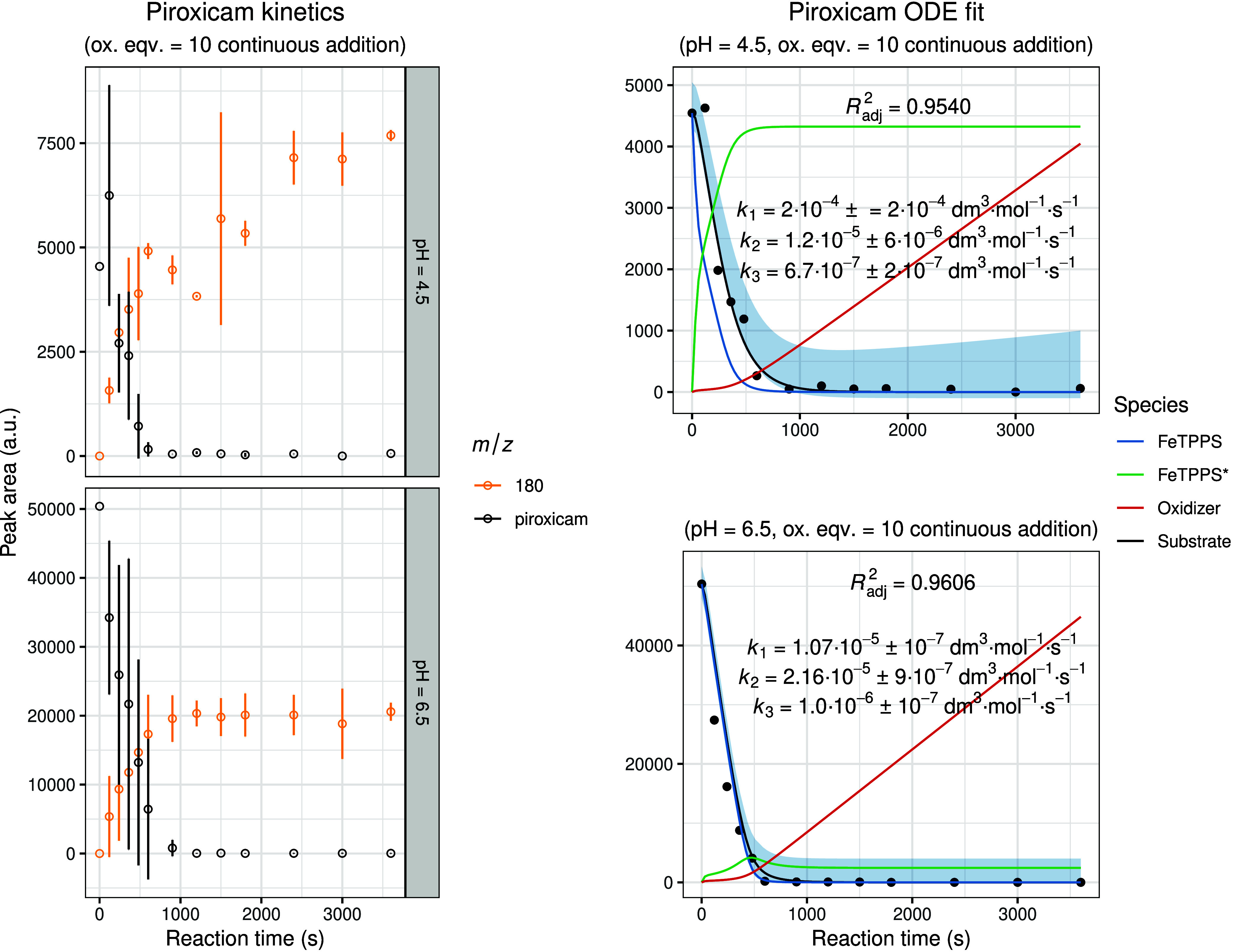
Experimental and modeled biomimetic oxidation kinetics of piroxicam. The left panel shows the experimental kinetic data obtained from three independent parallel experiments (mean ± SD), illustrating the time-dependent change in chromatographic peak areas for the substrate and identified oxidation metabolites. The right panel presents the corresponding fitted kinetic model together with simulated 95% confidence intervals (shaded regions). The calculated concentration–time profiles of FeTPPS and activated FeTPPS* (scaled by × 10), as well as the oxidizing agent (scaled by × 0.1), are included to visualize the dynamic behavior of all reactive species within the system.

Figure 11 summarizes the diagnostics of the ODE fits across all drug–pH combinations. The standardized residuals plotted against fitted values showed essentially the same pattern as the residuals plotted against time, and no pronounced systematic trends were apparent, suggesting that the model captured the overall kinetic profiles reasonably well. Nevertheless, higher response values were consistently associated with larger relative deviations, indicating residual heteroscedasticity. The correlation matrices of the fitted parameters, shown for *k*_1_–*k*_3_ only to enable complete comparison across all fits, also revealed that a subset of fits suffered from substantial parameter interdependence, accompanied by unusually large standard errors. This points to practical identifiability problems arising primarily from limited data informativeness, especially when the early part of the kinetic profile was not adequately captured, rather than from obvious structural misspecification of the model itself. In most cases these diagnostics remained acceptable, indicating that the model was able to distinguish data sets with sufficiently informative kinetic structure from those in which reliable parameter estimation was not feasible. Approximate 95% confidence intervals for fitted parameters may be obtained on the log scale as log*θ̂* ± 1.96 · *SE*_log*θ̂*_, followed by back-transformation to the original scale; this is the appropriate approach when estimation is performed on the log-parameter scale, as it preserves parameter positivity.

**Figure 11:**
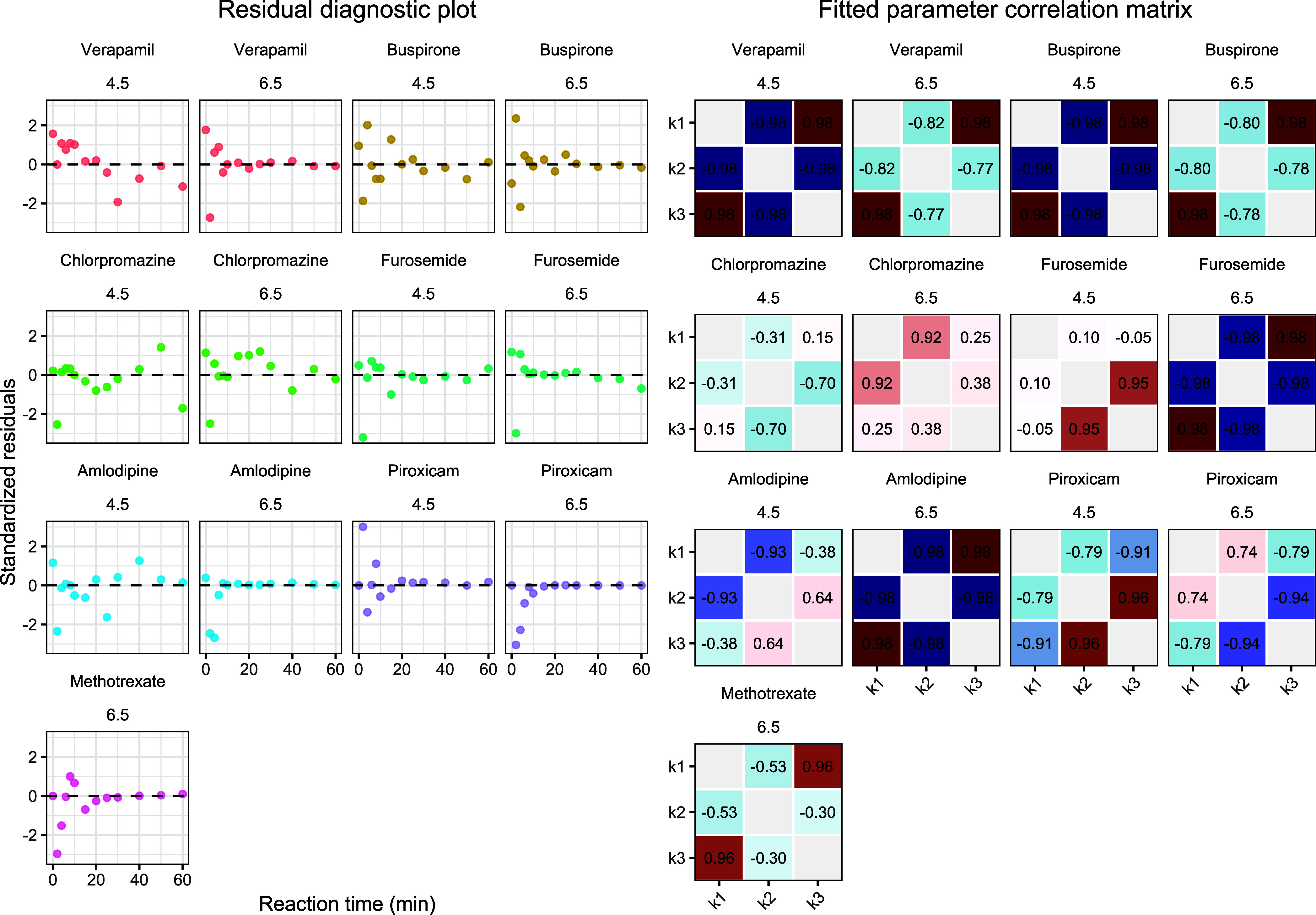
Model diagnostics for the individual ODE fits across all drug–pH combinations. Left: standardized residuals plotted against reaction time for each fit, faceted by drug and pH, to assess overall fit quality and detect systematic bias or heteroscedasticity. Right: within-fit correlation matrices of the estimated kinetic parameters; stronger shading highlights absolute parameter correlations above 0.9, indicating pronounced parameter interdependence and potential identifiability constraints.

### 3.4 Comparison of Kinetic Constants

To compare the reactivity patterns of the studied compounds, the kinetic constants obtained from the model fits were analyzed across all data sets. The fitted parameters (*k*_1_, *k*_2_, *k*_3_, compiled in Table 1) were first evaluated for internal consistency within each compound and pH condition. The majority of the data showed acceptable fitting uncertainty and reproducible values across the three parallel experimental runs, confirming the robustness of the kinetic modeling approach. In some instances where kinetic profiles were not clearly discernible (typically in the pH 4.5 cases) standard errors were comparable in magnitude to the kinetic constants.

**Table 1:** Summary of Fitted Kinetic Parameters for the Biomimetic Oxidation of the Studied Compounds under Two Different pH Conditions (except for Methotrexate at pH 4.5 Which Could Not Be Fitted)^a^

compound	pH	*k*_1_	*SE*(*k*_1_)	*k*_2_	*SE*(*k*_2_)	*k*_3_	*SE*(*k*_3_)	*CL*	*k*_2_/*CL*
verapamil	4.5	1.3 × 10^–4^	3 × 10^–4^	4.5 × 10^–4^	2 × 10^–4^	6.0 × 10^–5^	1 × 10^–4^	12.74	4.0 × 10^–5^
buspirone	4.5	4.0 × 10^–5^	2 × 10^–3^	8.0 × 10^–4^	3 × 10^–2^	2.0 × 10^–5^	9 × 10^–4^	0.79	9.0 × 10^–4^
chlorpromazine	4.5	2.8 × 10^–5^	3 × 10^–6^	2.8 × 10^–6^	9 × 10^–8^	6.1 × 10^–6^	9 × 10^–8^	13.37	2.0 × 10^–7^
furosemide	4.5	1.0 × 10^–4^	6 × 10^–6^	6.0 × 10^–6^	9 × 10^–6^	1.7 × 10^–5^	6 × 10^–7^	0.08	8.0 × 10^–5^
methotrexate	4.5							0.15	
amlodipine	4.5	1.8 × 10^–4^	1 × 10^–5^	9.8 × 10^–8^	5 × 10^–9^	1.9 × 10^–5^	2 × 10^–6^	2.83	3.0 × 10^–8^
piroxicam	4.5	2.0 × 10^–4^	2 × 10^–4^	1.2 × 10^–5^	6 × 10^–6^	6.7 × 10^–7^	2 × 10^–7^	1.14	1.0 × 10^–5^
verapamil	6.5	6.2 × 10^–6^	8 × 10^–7^	2.8 × 10^–5^	1 × 10^–5^	6.6 × 10^–7^	8 × 10^–8^	12.74	2.0 × 10^–6^
buspirone	6.5	2.8 × 10^–6^	6 × 10^–7^	1.8 × 10^–6^	4 × 10^–7^	3.7 × 10^–7^	8 × 10^–8^	0.79	2.0 × 10^–6^
chlorpromazine	6.5	3.0 × 10^–5^	6 × 10^–7^	3.0 × 10^–6^	7 × 10^–6^	8.6 × 10^–6^	8 × 10^–7^	13.37	2.0 × 10^–7^
furosemide	6.5	3.0 × 10^–4^	2 × 10^–4^	4.0 × 10^–6^	5 × 10^–6^	6.0 × 10^–5^	3 × 10^–5^	0.08	5.0 × 10^–5^
methotrexate	6.5	3.4 × 10^–6^	8 × 10^–7^	3.0 × 10^–5^	5 × 10^–5^	3.4 × 10^–7^	8 × 10^–8^	0.15	2.0 × 10^–4^
amlodipine	6.5	2.0 × 10^–4^	2 × 10^–4^	1.0 × 10^–3^	3 × 10^–3^	3.0 × 10^–5^	2 × 10^–5^	2.83	4.0 × 10^–4^
piroxicam	6.5	1.1 × 10^–5^	1 × 10^–7^	2.2 × 10^–5^	9 × 10^–7^	1.0 × 10^–6^	1 × 10^–7^	1.14	2.0 × 10^–5^

a Listed are the fitted rate constants (in L·mol^–1^·s^–1^) together with their standard errors (SE). Literature values of intrinsic clearance (CL in L·h^–1^·kg^–1^) are included for comparison, and the derived normalized parameter (*k*_2_/CL) illustrates the proportionality between catalytic turnover and substrate clearance.

When comparing the rate constants among the compounds, clear trends emerged. The substrate oxidation constant *k*_2_ varied most strongly between substrates, reflecting differences in their susceptibility toward metalloporphyrin-mediated oxidation. In contrast, the activation rate constant *k*_1_ and the catalyst degradation rate constant *k*_3_ remained within a confined range, indicating that FeTPPS stability was largely unaffected by substrate identity under the chosen experimental conditions. For compounds where the extended model was required, the noncatalytic oxidation constant *k*_4_ was typically two to 3 orders of magnitude smaller than *k*_2_, confirming that the direct oxidation pathway represented only a minor contribution to the overall reaction. Nevertheless, its inclusion improved the description of certain kinetic profiles exhibiting biphasic substrate decay.

A summary of the fitted kinetic constants is presented in Figure 12, where the values are plotted as a function of pH and compound identity. For most substrates, increasing the pH from 4.5 to 6.5 resulted in a decrease in the apparent oxidation rate constant (*k*_2_), although several compound-specific deviations are observed. In line with prior mechanistic studies on water-soluble iron­(III) porphyrins,^16^ this trend can be rationalized by a pH-dependent shift in the nature of the active oxidizing species. Under more acidic conditions, peroxide activation is expected to favor formation of a high-valent iron­(IV)–oxo porphyrin π-cation radical, which is capable of a rapid, concerted two-electron oxidation of suitable substrates.^16^ At higher pH, deprotonation equilibria and redox level crossing are anticipated to stabilize iron­(IV)–oxo species lacking porphyrin radical character, which are comparatively less oxidizing and more likely to engage in stepwise one-electron pathways. The combined influence of oxidant speciation, substrate ionization, and electrostatic interactions therefore provides a consistent framework for the generally lower *k*_2_ values observed at pH 6.5, as well as for the presence of substrate-dependent outliers. In Figure 12, the normalized kinetic parameter (*k*_2_/*CL*) is also presented for the available compound–pH combinations. Not all compounds and pH conditions are shown in this comparison: data sets for which the partial differential equation (PDE) simulations indicated rapidly varying concentrations of the activated catalyst species (FeTPPS*) were excluded, in order to maintain the validity of the steady-state assumptions underlying the normalization analysis. The resulting *k*_2_/*CL* values remain relatively constant across the data set, which is expected from the proportionality relationship between the apparent oxidation rate constant and the intrinsic clearance (*CL*) of the substrate. Intrinsic clearance values were taken from the literature^17–23^ and are expressed in L h^–1^ kg^–1^ when reported in body-weight–normalized units. When literature values were reported per milligram of protein, a scaling factor of 0.07 was applied, based on standard microsomal protein per gram of liver and liver weight to body weight ratios (for humans taken as 40–50 mg_protein_ g_liver_ and 21–25 g_liver_ kg_weight_, respectively). Intrinsic clearance is widely regarded as the gold-standard metric for assessing hepatic metabolic capacity, as liver microsomes are enriched in cytochrome P450 enzymes, which dominate oxidative drug metabolism. Importantly, the cytochrome P450 active site shares a high degree of structural and functional similarity with metalloporphyrin catalysts, providing a strong mechanistic basis for comparison with the present biomimetic system.^1,3^

**Figure 12:**
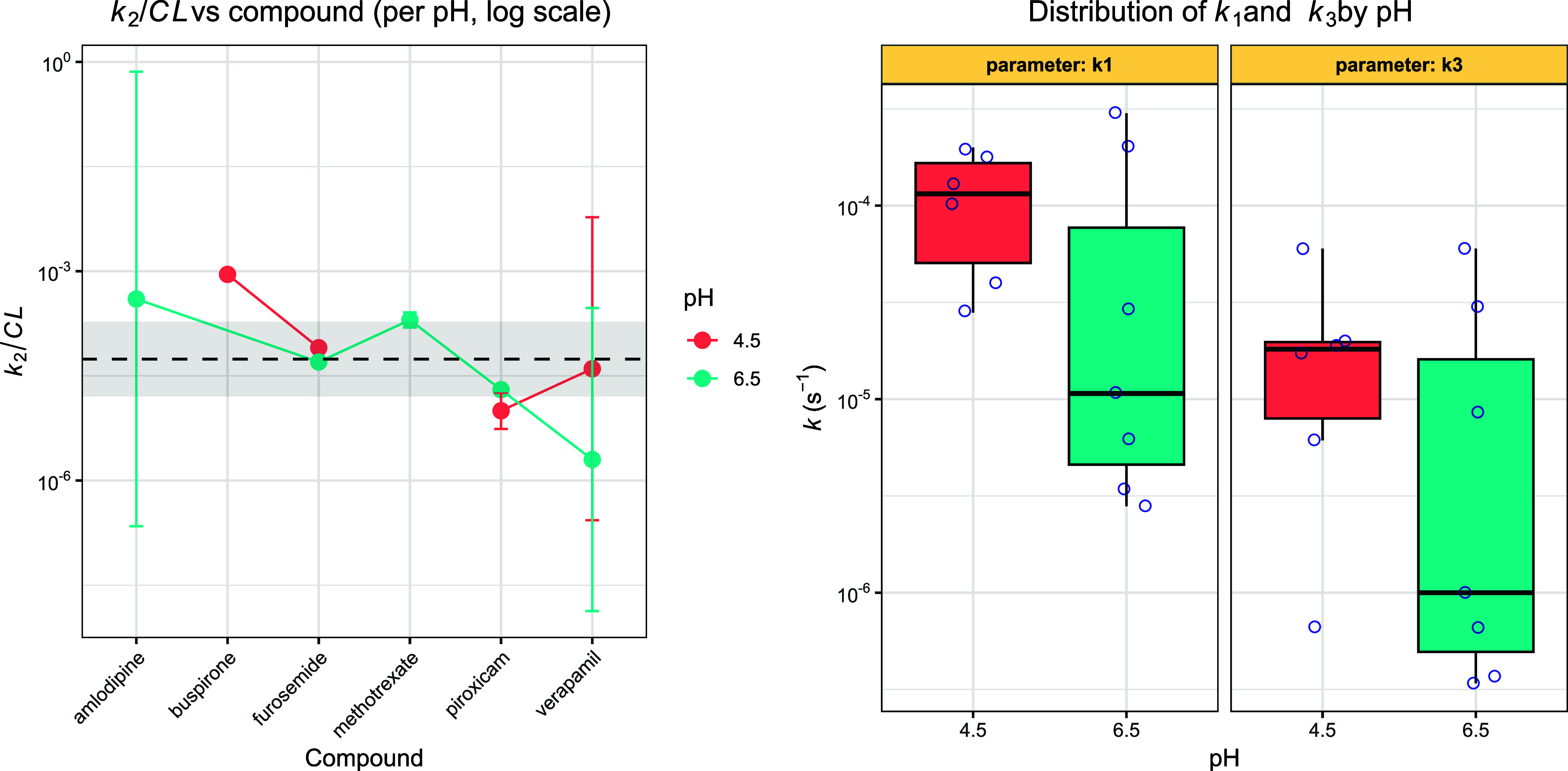
Comparison of kinetic constants across the studied compounds and pH conditions. The normalized parameter (*k*_2_/CL) is shown on the left, illustrating its relative constancy across the data set in agreement with the proportionality law derived from the catalytic rate expression. Mean value and 95% confidence bands are shown. On the right the pooled *k*_1_ and *k*_3_ values are shown to fall within a similar order of magnitude range and display consistent relative magnitudes at both pH 4.5 and 6.5, reflecting the similar balance between catalyst activation and deactivation processes under these conditions.

This proportionality arises from the steady-state approximation of the catalytic cycle, where the overall rate of substrate oxidation can be expressed as follows. At low substrate concentrations ([S] ≪ *K*_M_), intrinsic clearance in classical enzyme kinetics is

$$CL_{\mathrm{int}}=\frac{v_{\max}}{K_{M}}=\frac{k_{\mathrm{cat}}{\left[E\right]}_{\mathrm{Total}}}{K_{M}}$$ 7

showing that *CL*_int_ is proportional to the catalytic efficiency *k*_cat_/*K*_M_ and to the available enzyme abundance [E]_Total_. In the present kinetic scheme, the observable oxidation rate of the substrate is given by

$$v=k_{2}\left[S\right]\left[{\mathrm{FeTPPS}}^{*}\right]$$ 8

By the pharmacokinetic definition of clearance under these conditions,

$$CL=\frac{v}{\left[S\right]}=k_{2}\left[{\mathrm{FeTPPS}}^{*}\right]$$ 9

and therefore,

$$\frac{k_{2}}{CL}=\frac{1}{\left[{\mathrm{FeTPPS}}^{*}\right]}$$ 10

Equations 7–10 are consistent when the concentration of the kinetically competent catalyst species is identified with the effective enzyme abundance, [E]_Total_ ≈ [FeTPPS*], and when the elementary oxidation step constant is interpreted as the turnover number *k*_2_ ≈ *k*_cat_. Under conditions where [FeTPPS*] varies slowly in time, the ratio *k*_2_/*CL* is therefore expected to show minimal variation across substrates and pH values, in agreement with the observed results. The *k*_2_/*CL* values also display similar relative trends between the two pH conditions, indicating that substrate-specific effects are largely compensated when normalized to intrinsic clearance.

In Figure 12, the rate constants *k*_1_ and *k*_3_ are also shown, pooled across all compounds for the two pH conditions. These constants are theoretically independent of substrate identity, and indeed their values fall within the same order of magnitude across the data set, although substantial experimental variability is observed. Notably, the relative magnitudes of *k*_1_ and *k*_3_ remain consistent under both pH conditions, reflecting the stable relationship between catalyst activation and deactivation kinetics in the metalloporphyrin system.

## 4 Discussion

The kinetics of biomimetic oxidation catalyzed by a metalloporphyrin complex were investigated under a range of experimental conditions to identify parameters yielding reliable and interpretable kinetic data. Systematic variation of oxidizing agent amounts, pH, and mode of oxidant addition allowed determination of the optimal conditions for kinetic analysis. Among these, the use of ten equivalents of oxidizing agent and continuous addition proved most suitable, providing gradual reaction progress and avoiding the rapid, uncontrolled oxidation characteristic of one-shot addition experiments. Kinetic modeling based on a mechanistic system of ordinary differential equations was successfully applied to both one-shot and continuous oxidant delivery modes, accurately reproducing the experimental data. The model accounted for catalyst activation, substrate oxidation, and catalyst deactivation, and was extended in specific cases to include noncatalytic oxidation pathways. The fitted kinetic profiles showed good accordance between experimental and simulated data across all compounds tested.

The extended data set across substrates and pH conditions showed that experiments at pH 6.5 generally produced more pronounced and reproducible kinetic traces and, in most cases, higher overall substrate conversion than at pH 4.5. Rather than implying faster single-step oxidation at higher pH, this behavior is more consistent with a pH-dependent shift in the reactive iron–oxo manifold and catalyst lifetime. Mechanistic studies on water-soluble Fe­(III) porphyrins indicate that more acidic conditions favor formation of an oxo-Fe­(IV) porphyrin π-cation radical (“Compound I–type”), whereas at higher pH the system increasingly stabilizes oxo-Fe­(IV) species lacking porphyrin radical character.^16^ In peroxide-driven FeTPPS chemistry, highly oxidizing intermediates and redox cycling can also accelerate unproductive catalyst oxidation, reducing trace reproducibility and limiting conversion despite initially fast kinetics.^24,25^ The fitted substrate-specific kinetic constants demonstrated good proportionality with literature-reported intrinsic clearance (*CL*) values, confirming that the biomimetic oxidation system reflects physiologically relevant liver-based oxidative drug metabolism. In contrast, the catalyst-dependent rate constants (*k*_1_ and *k*_3_), representing activation and deactivation, respectively, were largely substrate-independent and remained within the expected magnitude range for metalloporphyrin-based systems.

When the fitted *k*_2_ values are compared with estimated charge states (Table 2; p*K*_a_ values taken from ref 26), no single, monotonic electrostatic relationship emerges across all substrates. While a decrease in *k*_2_ at pH 6.5 is credibly observed for verapamil, several other comparisons are statistically weak because the fitted uncertainties at one pH (notably buspirone at pH 4.5 and amlodipine at pH 6.5) are larger than, or comparable to, the point estimates. In addition, piroxicam shows a reproducible increase in *k*_2_ at pH 6.5 despite becoming more negatively charged, indicating that substrate ionization can alter intrinsic reactivity in ways that are not captured by a purely Coulombic “attraction/repulsion” model. Overall, the data are more consistent with pH modulating *k*_2_ through a combination of (i) pH-dependent changes in the iron–oxo oxidant manifold (Compound I–type versus Fe­(IV)=O without porphyrin radical character), (ii) substrate speciation effects on the preferred one-electron vs two-electron pathway, and (iii) fit identifiability limitations for specific substrates under certain conditions. In this framework, pH 4.5 is expected to favor more “Compound I–like” reactivity (formally capable of net two-electron oxidation), whereas pH 6.5 increasingly stabilizes less oxidizing Fe­(IV)=O species that bias oxidation toward slower, stepwise electron-transfer or PCET pathways. Concomitantly, both the apparent activation (*k*_1_) and deactivation (*k*_3_) rate constants are expected to decrease at higher pH, reflecting slower peroxide activation but also reduced catalyst self-oxidation. The observed improvement in conversion and reproducibility at pH 6.5 is therefore consistent with enhanced catalyst longevity and more controlled redox cycling, rather than universally larger *k*_2_ values.

**Table 2:** Summary of the Relationship between Acid–Base Character (A: Acid; B: Base), Formal Charge (Calculated from Literature pK_a_ Values), and the Fitted Substrate Oxidation Rate Constant (*k*_2_) under Two Different pH Conditions^a^

compound	character	charge (pH 4.5 → 6.5)	*k*_2_ (pH 4.5)	*k*_2_ (pH 6.5)
buspirone	BB	+1 → + 0.9	*7 × 10* ^*–4*^ *± 3 × 10* ^*–2*^	**1.8 × 10** ^**–6**^ **± 4 × 10** ^**–7**^
verapamil	B	+1 → 0	5 × 10^–4^ ± 2 × 10^–4^	3 × 10^–5^ ± 1 × 10^–5^
chlorpromazine	B	+1 → 0	**2.8 × 10** ^**–6**^ **± 9 × 10** ^**–8**^	3 × 10^–6^ ± 7 × 10^–6^
amlodipine	B	+1 → 0	**9.8 × 10** ^**–8**^ **± 5 × 10** ^**–9**^	1 × 10^–3^ ± 3 × 10^–3^
furosemide	AA	–0.89 → – 1.0	6 × 10^–6^ ± 9 × 10^–6^	4 × 10^–6^ ± 5 × 10^–6^
piroxicam	AB	–0.14 → – 0.94	**1.2 × 10** ^**–5**^ **± 6 × 10** ^**–6**^	**2.2 × 10** ^**–5**^ **± 9 × 10** ^**–7**^
methotrexate	AAB	–1.9 (only at pH 6.5)		3 × 10^–5^ ± 5 × 10^–5^

a Values with high uncertainty are indicated in italics, moderately uncertain values in plain text, and values with higher certainty in bold.

Future studies could further clarify these charge-dependent effects by directly monitoring catalyst-substrate interactions under varying protonation states. In particular, *in situ* spectroscopic techniques such as Raman, UV–vis or NMR spectroscopy could be employed to detect changes in the electronic environment of FeTPPS during substrate binding and oxidation, thereby providing direct experimental evidence for the proposed electrostatic modulation of catalytic efficiency. Although the current kinetic model effectively captured the main features of the reaction, certain limitations remain. The experimental temporal resolution could be improved by acquiring data at shorter time intervals, particularly during the initial phase of oxidant addition. Expanding the pH range would further clarify mechanistic variability associated with protonation states of the catalyst or substrate. Deviations of the obtained rate constants from theoretical expectations likely stem from experimental uncertainties or simplifications inherent to the model assumptions.

## 5 Conclusions

The present study demonstrates that biomimetic oxidation kinetics mediated by a metalloporphyrin catalyst can be effectively modeled and quantified under controlled experimental conditions. The developed kinetic model, based on a mechanistically interpretable system of ordinary differential equations, successfully described the activation, catalytic oxidation, and deactivation steps of the FeTPPS catalyst. Across a series of structurally diverse compounds and multiple pH conditions, the model provided consistent fits and meaningful rate constants, with pH 6.5 emerging as the condition supporting the most efficient and reproducible oxidation behavior. Importantly, continuous addition of the oxidizing agent proved advantageous over one-shot addition, as it prevented rapid catalyst deactivation, enabled time-resolved kinetic observation, and allowed reliable parameter estimation within the developed modeling framework. Comparison of the fitted kinetic parameters with intrinsic clearance data from the literature revealed a clear proportional relationship between the catalytic oxidation rate constant and substrate clearance, thereby supporting the biomimetic relevance of the metalloporphyrin system as a mechanistic model of cytochrome P450–mediated oxidation. Notably, the intrinsic clearance values used for comparison were derived from liver microsomal assays, which are widely regarded as the gold standard for quantifying hepatic metabolic capacity and for modeling *in vivo* liver metabolism. The observed agreement therefore underscores the physiological relevance of the present biomimetic approach. Overall, this work establishes a robust experimental–computational framework for the quantitative analysis of biomimetic oxidation kinetics within a mechanistic CYP enzyme modeling context. By linking catalyst-level kinetic parameters with pharmacokinetic observables, the approach provides a practical bridge between chemical catalysis and drug metabolism and can be readily extended to other catalytic systems or oxidant delivery regimes. Future studies integrating real-time spectroscopic monitoring of metalloporphyrin species are expected to further enhance mechanistic insight and improve the predictive power of the kinetic model.

## Supplementary Material



## Notes

Files include data.csv: raw measurement data; analysis.R: code for statistical analysis of the data and generating plots (ZIP)
