# Supplementary material for: Kinetic Study of Metalloporphyrin-Based Biomimetic Oxidation of Drugs Using One-Shot and Continuous Oxidizing Agent Addition Modes
Source: J Phys Chem B. 2026 Apr 28;130(18):4822–37. doi: 10.1021/acs.jpcb.6c00851 (PMC13158922; doi:10.1021/acs.jpcb.6c00851)
Supplement: Supplementary file 1 [file jp6c00851_si_001.zip › Supplementary Information.pdf]

Kinetic Study of Metalloporphyrin-based Biomimetic Oxidation of Drugs Using One-Shot and Continuous Oxidizing Agent Addition Modes

Balázs Csillag[‡,1,2], Ernák Ferenc Várda[‡,3], Anna Kamilla Kis[3], Zsombor Máté Hámor[3], Zsombor Márton Mohácsi[1,2], Anna Vincze[1,2], András Dénes Marton[3,4], Balázs Decsi[3], Diána Balogh-Weiser[5,6,7], Balázs Volk[3,8], Arash Mirzahosseini[1,2,*], and György Tibor Balogh[1,2,3,*]

[‡] These authors contributed equally to this work.

[1] Department of Pharmaceutical Chemistry, Semmelweis University, Budapest, H-1092, Hungary
[2] Center for Pharmacology and Drug Research & Development, Semmelweis University, Budapest, H-1085, Hungary
[3] Department of Chemical and Environmental Process Engineering, Budapest University of Technology and Economics, Budapest, H-1111, Hungary
[4] Ambimass LLC, Budapest, H-1031, Hungary
[5] Department of Physical Chemistry and Materials Science, Budapest University of Technology and Economics, Budapest, H-1111, Hungary
[6] Department of Organic Chemistry and Technology, Budapest University of Technology and Economics, Budapest, H-1111, Hungary
[7] Spinsplit LLC, Vecsés, H-2220, Hungary
[8] Directorate of Drug Substance Development, Egis Pharmaceuticals PLC, Budapest, H-1106, Hungary

[*] Correspondence: Arash Mirzahosseini <mirzahosseini.arash@semmelweis.hu>, György Tibor Balogh <balogh.gyorgy.tibor@semmelweis.hu>

Files included:

`data.csv` contains the study dataset used for all statistical analyses and figure generation. When read by **`analysis.R`**, it enables full reproduction of the results reported in the study.
